# Overcoming Individual Limitations Through Distributed Computation: Rational Information Accumulation in Multigenerational Populations

**DOI:** 10.1111/tops.12596

**Published:** 2022-01-15

**Authors:** Mathew D. Hardy, Peaks M. Krafft, Bill Thompson, Thomas L. Griffiths

**Affiliations:** ^1^ Department of Psychology Princeton University; ^2^ Creative Computing Institute University of the Arts London; ^3^ Department of Computer Science Princeton University

**Keywords:** Bayesian inference, Cultural evolution, Collective intelligence, Distributed computation, Group rationality, Social learning

## Abstract

Many of the computational problems people face are difficult to solve under the limited time and cognitive resources available to them. Overcoming these limitations through social interaction is one of the most distinctive features of human intelligence. In this paper, we show that information accumulation in multigenerational social networks can be produced by a form of distributed Bayesian inference that allows individuals to benefit from the experience of previous generations while expending little cognitive effort. In doing so, we provide a criterion for assessing the rationality of a population that extends traditional analyses of the rationality of individuals. We tested the predictions of this analysis in two highly controlled behavioral experiments where the social transmission structure closely matched the assumptions of our model. Participants made decisions on simple categorization tasks that relied on and contributed to accumulated knowledge. Success required these microsocieties to accumulate information distributed across people and time. Our findings illustrate how in certain settings, distributed computation at the group level can pool information and resources, allowing limited individuals to perform effectively on complex tasks.

## Abstract 2

**Blurb**: Many of the problems people face are difficult to solve under the limited time and resources available to them. We show that individuals can overcome these limitations by following a simple social learning heuristic that yields distributed Bayesian inference at the population level. We test our model in two large behavioral experiments, comparing observed knowledge accumulation with the Bayesian ideal in multigenerational microsocieties.

## 1 Introduction

Humans' success and survival depends on our ability to make intelligent choices and judgments. Like other animals, we must do so under hard constraints on the resources available to make our decisions (Griffiths, 2020). Not only is our cognition constrained by our restricted brain capacity, but we also must learn from limited and often incomplete data. This is true not only in our finite childhoods and lifespans but also in the limited opportunities we have to learn many crucial facts—to survive, we need to learn about what life‐threatening dangers exist in our immediate environments (from chemicals to electric outlets to poisonous insects and animals).

Given these constraints, understanding how people routinely form accurate beliefs on complex topics is a central focus of research in cognitive science, psychology, and economics (Simon, 1990). However, people rarely develop beliefs alone—rather, they learn from the knowledge, experiences, and opinions of other people. By doing so, individuals can obtain useful information while expending little physical or cognitive effort. A particularly important focus of previous research has been on the effects of social learning when repeated over successive groups of individuals, such as child–parent learning, formal education, and other domains where knowledge is transmitted from older members of a popvvvulation to younger learners. In these multigenerational settings, knowledge can accumulate over time in a population, allowing individuals to extend their cognitive skills by learning from others (see, e.g., Almaatouq, Alsobay, Yin, & Watts, 2021; Almaatouq et al., 2020; Belikov, Rzhetsky, & Evans, 2020; Caldwell, Atkinson, & Renner, 2016; Frey & Goldstone, 2018; Galesic, Olsson, Dalege, van der Does, & Stein, 2021; Goldstone, Wisdom, Roberts, & Frey, 2013; Hazła, Jadbabaie, Mossel, & Rahimian, 2021; Kempe & Mesoudi, 2014b; Mesoudi, 2016; Mesoudi & Thornton, 2018; Miton & Charbonneau, 2018; Riedl, Kim, Gupta, Malone, & Woolley, 2021; Rzhetsky, Foster, Foster, & Evans, 2015; Salhab, Ajorlou, & Jadbabaie, 2020; Wisdom, Song, & Goldstone, 2013; Wojtowicz & DeDeo, 2020, for recent overviews and related studies). Crucially, the constraints and structure of interpersonal transmission often lead collective knowledge and learning to differ from individual outcomes (Kirby, Tamariz, Cornish, & Smith, 2015; Ravignani, Thompson, Grossi, Delgado, & Kirby, 2018; Silvey, Kirby, & Smith, 2019).

The accumulation of collective knowledge through sequential social learning is known as *cultural transmission* (Boyd & Richerson, 1985) and is thought to underpin cumulative cultural evolution (Mesoudi, 2011). Cultural transmission has been studied experimentally in a number of paradigms, including the evolution of simple technologies such as knots (Muthukrishna, Shulman, Vasilescu, & Henrich, 2014), virtual fishing nets (Derex, Beugin, Godelle, & Raymond, 2013), stone tools (Morgan et al., 2015), or arrowheads (Mesoudi & O'Brien, 2008); artificial languages (Kirby, Cornish, & Smith, 2008); jigsaw puzzles (Kempe & Mesoudi, 2014a); and social phenomena like stereotypes (Martin et al., 2014).

Theories of cultural evolution have primarily been underpinned by a Darwinian framework grounded in parallels and disanalogies with biological evolution (Boyd & Richerson, 1985; Mesoudi & Whiten, 2008; Nettle, 2020; Smolla et al., 2020). These frameworks have shown that the accumulation of knowledge and technology can be understood as a form of evolution, helping to situate cultural evolution within the biological and evolutionary sciences (Laland, Sterelny, Odling‐Smee, Hoppitt, & Uller, 2011). While successful, evolutionary frameworks have been difficult to connect to psychological theory (Heyes, 2018) and are therefore difficult to connect with the concepts of computation that are central to the study of cognition and intelligence (Nettle, 2020). Similarly, while research on collective intelligence has extensively documented the advantages of groups over individuals, the computational structure of the social processes that lead to long‐term, open‐ended collective intelligence remain unclear (Krafft et al., 2016).

One way to measure the accumulation of knowledge in populations is by viewing collective behavior as distributed computation, a process that allows groups to “store and process the cumulative innovations and collaborations of generations of individuals” (Smaldino & Richerson, 2013). From this perspective, knowledge accumulation becomes a problem of *distributed* Bayesian inference, extending probabilistic models of inference in individuals to the group setting (Chater, Tenenbaum, & Yuille, 2006; Griffiths, Chater, Kemp, Perfors, & Tenenbaum, 2010; Harper, 2009). This perspective establishes a formal connection between cultural evolution and statistical models of social learning used in cognitive science (Cushman & Gershman, 2019) and economics (Acemoglu, Dahleh, Lobel, & Ozdaglar, 2011).

In this paper, we build on this work and show how social learning can facilitate rational action that goes beyond the direct experience of individuals. That is, we show how social interactions allow limited individuals to improve their cognition without modifying their time and resource constraints. We do this in part by offering a formal criterion for *population rationality*: the probability that any individual in a network makes a particular decision is the same as the probability of that decision under a Bayesian posterior distribution conditioned on *all* the information observed by the population (Foster, 2018, cf.).

To develop our account, we first formulate a model of individual social learning based on a simple heuristic that requires only limited social observation. We then show that under certain conditions, groups of individuals following this heuristic will accumulate information through distributed Bayesian inference. This model offers insight into how social learning can extend the cognitive abilities of limited individuals—while conditioning on all accumulated information would be too complex for any individual, social interactions allow individuals to benefit from this information while expending little cognitive effort. That is, population rationality can be achieved by networks of highly limited individuals.

We tested our model in two large‐scale experiments where participants made basic categorization decisions in simple multigenerational networks. In both experiments, the decisions of participants at one generation were propagated to those in the next generation, allowing us to study belief accumulation and transmission in a controlled laboratory setting. We found that a substantial proportion of the data from these experiments are well‐approximated by our model. Furthermore, by comparing participants' choices with the Bayesian posterior distribution conditioned on the information observed by the entire population, we were able to quantify accumulation in our experimental networks relative to the Bayesian ideal. Our findings thus offer a clear demonstration of how limited individuals can use simple social learning heuristics to make intelligent inferences.

## 2 Information accumulation through social sampling

Here, we describe a simple social learning heuristic we call *social sampling* that yields distributed Bayesian inference at the population level. That is, populations of individuals following social sampling will accumulate information in a way that is consistent with Bayesian inference, allowing individuals to offload computation to the group and make accurate inferences with little cognitive effort. We focus on multigenerational settings, where individuals are organized in discrete “batches” and learn from those in the previous generation. We also assume individuals observe others' true beliefs and do not need to discard or modify any observations.

### 2.1 Model

Information accumulation by rational agents is specified by Bayes' rule, which indicates how a probability distribution over hypotheses θ (known as the *prior* distribution) should be updated (to the *posterior* distribution) in light of evidence D:

(1) $$p\left(\theta |D\right)=\frac{p(D|\theta )\cdot p(\theta )}{p(D)}.$$

In this paper, we extend this characterization of optimal belief updating from individuals to groups (see Chater, Oaksford, Hahn, & Heit, 2010; Griffiths, Kemp, & Tenenbaum, 2008; Tenenbaum, Kemp, Griffiths, & Goodman, 2011, for reviews of Bayesian models of individual cognition). Viewing a population as a single agent is a perspective with roots in many traditions, including philosophy (Easwaran, 2019), economics (Gale & Kariv, 2003), economics (Hayek, 1945), organization science (Argote, 2012), cognitive science (Krafft, Shmueli, Griffiths, Tenenbaum, & Pentland, 2021), anthropology (Hutchins, 1995), collective intelligence (Engel et al., 2015; Woolley, Chabris, Pentland, Hashmi, & Malone, 2010), ethology (Sasaki & Pratt, 2011), and computer science (Lynch, 1996; Shoham & Leyton‐Brown, 2009). Crucially, as we will show, rational action at the population level requires only limited computation by individuals—while each individual agent may follow a simple heuristic, the information accumulated by the population as a whole makes it possible for those individuals to act rationally.

We consider the simple case where individuals reason about a set of that can be either true or false, represented as a binary feature vector x. Individuals beliefs are shaped by pieces of evidence $Y=(y_{1},\text{…},y_{T})$ that arrive over time. At each time t, individuals observe J pieces of information about feature i, with $P_{i}=P\left(y_{ijt}=1|x_{i}=0\right)=\theta_{i0}$ if $x_{i}=0$ and $P_{i}=P\left(y_{ijt}=1|x_{i}=1\right)=\theta_{i1}$ if $x_{i}=1$. For simplicity, we let $S_{\mathrm{it}}=\sum_{j=1}^{J}1\left(y_{\mathrm{ijt}}=1\right)$ be the number of positive observations made about feature i at time t.

We assume that the popularity of a belief among members of the population acts as a prior distribution over beliefs for new individuals drawing inferences about the environment. Specifically, an individual a first chooses a member of the population $a^{\prime }$ to learn from uniformly at random.^1^ The learner a then accepts or rejects their companion's decision $d_{a^{\prime },i,t-1}$ with probability proportional to ${\left(\theta_{i,d_{a^{\prime },i,t-1}}\right)}^{S_{it}}{\left(1-\theta_{i,d_{a^{\prime },i,t-1}}\right)}^{J-S_{it}}$, the likelihood of the evidence that a observes at time t based on the beliefs of $a^{\prime }$.

In an infinite population of individuals following this strategy, the probability that an individual makes a categorization decision about feature i is equal to the posterior probability of that decision conditioned on the evidence observed by the *entire* population:

(2) $$p_{i,t+1}=P\left(x_{i}=1|y_{i,\cdot ,\leq t}\right).$$

This result establishes that social sampling is a valid algorithm for distributed Bayesian inference in infinitely large populations, satisfying our criterion for population rationality (see Supplementary Electronic Material (SEM) for proof). The finite population case is more complex, but social sampling can be seen to be formally equivalent to a class of sequential Monte Carlo algorithms known as particle filters (Crisan & Doucet, 2002; Murphy, 2012). Social sampling can thus support Bayesian inference in expectation in finite populations, allowing groups of bounded individuals to accumulate knowledge over time.

### 2.2 Measuring accumulation relative to optimal inference

The model we have outlined illustrates how a simple social decision‐making heuristic can lead to optimal information aggregation over generations. This suggests that in certain contexts the Bayesian posterior distribution can be used to construct a valid upper limit on information accumulation, because posterior distributions are information‐theoretically and decision‐theoretically optimal belief representations (Bernardo & Smith, 2000; Jaynes, 2003; Ortega, 2011). To quantify ideal information accumulation, we use the sufficient statistics of the Bayesian posterior distribution over the environment features given the evidence observed by the entire population. We assume a uniform prior, and so the posterior probability that a feature categorization is correct given the evidence received by the group up to generation t is

(3)

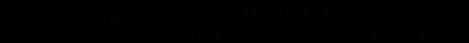

where $S_{i,\leq t}=\sum_{k=1}^{t}\sum_{j=1}^{J}1\left(y_{\mathrm{ijk}}=1\right)$ is the total positive evidence observed for feature i up to time t.

The sufficient statistics of this posterior distribution are $\frac{S_{i,\leq t}}{t\cdot J}$, or the proportion of pieces of evidence favoring a feature categorization. Because these statistics can be used to exactly compute the posterior distribution, we can measure accumulation in observed networks by comparing this statistic with the popularity of a belief, or proportion of people who believe it. We express this formally by letting $p_{\mathrm{it}}=\frac{1}{N}\sum_{a}1\left(d_{a,i,t-1}=1\right)$ be the proportion of individuals who believe $x_{i}=1$ at time t−1, with $d_{ait}$ indicating whether individual a at generation t chooses category i, and N representing the number of individuals in each generation. If the popularity is close to $\frac{S_{i,\leq t}}{t\cdot J}$ at time t, we can conclude that information is being effectively accumulated in the population.

### 2.3 Summary

This analysis demonstrates that individuals in multigenerational networks can improve their inferences by following a simple social learning heuristic. To perform social sampling, an individual first randomly selects a person in the previous generation to learn from. They then evaluate their companion's belief against observed evidence—the more aligned the belief with the evidence, the higher the chance the individual will accept it as their own, otherwise they will continue searching and sample another person. While similar social learning models have been explored in multiarmed bandit problems (Celis, Krafft, & Vishnoi, 2017; Krafft, 2017; Krafft et al., 2021), they have not been tested in controlled laboratory settings.

Social sampling is simple to perform and only requires limited computation by individuals. More precisely, at any time the popularity of a hypothesis will approximate the true Bayesian posterior probability conditioned on all the evidence observed by the population, and so the population itself performs distributed Bayesian inference. Because this distributed inference matches the inference problems individuals face, our model allows us to be precise about *how* social learning extends people's cognitive abilities.

In our model, individuals make unbiased judgments and cannot choose which information to transmit. Instead, every person's true beliefs can be observed by those in the next generation. While this setup—and assumptions of uniform sampling of others' true beliefs—is highly simplified, it reflects a common approach to modeling individual cognition as a two‐stage decision‐making process (Howard & Sheth, 1969; Krumme, Cebrian, Pickard, & Pentland, 2012; Payne, 1976; Pratt, Sumpter, Mallon, & Franks, 2005; Seeley & Buhrman, 1999; Vul, Goodman, Griffiths, & Tenenbaum, 2014) that has also been applied to modeling iterated learning in populations (Bonawitz, Denison, Gopnik, & Griffiths, 2014; Kalish, Griffiths, & Lewandowsky, 2007; Mozer, Pashler, & Homaei, 2008; Sanborn & Griffiths, 2008; Vul & Pashler, 2008) and allows us to make progress in understanding knowledge accumulation in a way that can be studied empirically and extended to more naturalistic settings.

While our model shows how group‐level Bayesian inference is possible, can we identify and quantify accumulation in real populations? To investigate, we ran two behavioral experiments where participants made categorization decisions in multigenerational networks. In both experiments, we assessed population rationality by comparing observed popularity with the Bayesian ideal and evaluated our social sampling model by comparing it with several alternative models.

## 3 Experiment 1: Gem classification

In Experiment 1, participants completed a categorization task that was framed as gemstone classification. This experiment was designed to closely mirror an idealized setting in which optimal information aggregation is possible through our proposed Bayesian social sampling mechanism.

**Fig. 1:**
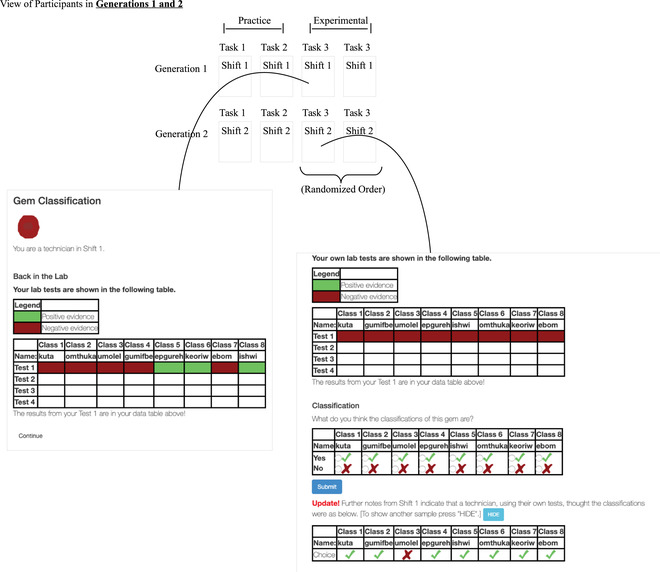
Participant interface for Experiment 1. Each categorization task consisted of a different set of gem classifications that were given random nonsense words. Each participant belonged to a “generation” of participants, called a “shift” in this experiment. In each generation after the first, participants could view the gem classifications made by participants in the last generation. Each participant made their own classification decisions for a gem four times after observing four pieces of non‐social evidence.

In this experiment, participants played the role of technicians classifying gemstones in a certain shift (i.e., generation).^2^ Each gemstone could have up to eight potential classifications, with each classification being a randomized nonsense word (e.g., “pesho,” “ivil,” “thyun”). On each trial, participants observed social information in the form of classification judgments drawn from the previous shift of technicians, as well as non‐social information in the form of a set of four new laboratory results. The laboratory results presented stochastic positive or negative evidence for each potential gemstone classification. We recruited three independent networks of participants, with each network including 10 shifts of 20 technicians (i.e., 200 participants per network). The interface for the experiment is illustrated in Fig. 1.

### 3.1 Methods

#### 3.1.1 Participants

We recruited 600 participants from Amazon Mechanical Turk. Recruitment was restricted to participants in the United States with an Amazon Mechanical Turk approval rating of 95 or above. We paid participants $1.75 as compensation, plus a performance‐based bonus payment of up to $0.50. The task typically took participants under 10 min to complete. Prior to starting the experiment, participants completed an attention and comprehension check which included questions about details of the study, including the probability of different kinds of evidence appearing in individual tests. Participants who failed to answer these questions correctly in three attempts were excluded from taking part in the study.

#### 3.1.2 Stimuli

On each round of the experiment, participants observed a cartoon gemstone positioned at the top of the screen on a white background. Each gem had eight possible binary classifications, and participants performed lab tests to make these classifications. Each test resulted in either positive or negative evidence for each classification. Fig. 1 shows an example participant view.

Classifications were given labels from an artificial vocabulary. These labels were different on each trial a participant completed (i.e., none of the labels recurred across trials for a participant) and were presented in randomized order. After each test, participants indicated which of the eight classifications they thought were true for this gem and could modify these classifications throughout the trial.

Participants in generations 2–10 observed the final classification decisions of a randomly sampled participant from the previous generation of the same network. Participants could choose to resample from the previous generation at any time during the trial. When participants chose to resample, the new sample was selected at random with replacement from the 20 participants in the previous generation of the same network. Social information (an earlier participant's classification decisions) was presented in a feedback table below the participant's own feedback table. After completing the fourth lab test, the results of the test were displayed and participants moved on to the next trial.

#### 3.1.3 Procedure

Participant completed four trials (i.e., gem classifications). The first two trials were practice rounds and were presented in the same order for all participants. The last two tasks were experimental trials and were ordered using simple randomization. We limited our analysis to data from the two experimental trials.

**Fig. 2:**
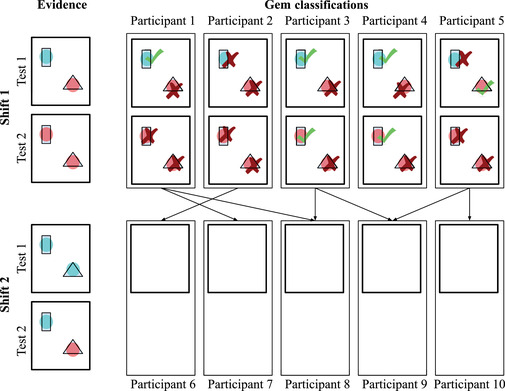
Illustration of the evidence correlation structure used in Experiment 1. If a gem classification test was positive for one participant, it was positive for all participants in that generation on that classification task. Positive evidence about a classification is illustrated here using turquoise circles and negative evidence with red circles. The classification judgments made by participants are shown as checkmarks and x‐marks. Participants could change their classifications after each new test.

Mirroring the social sampling model, participants were organized into discrete shifts, or “generations.” We recruited three independent networks of participants, with each network consisting of 10 generations with 20 participants per generation (see Fig. 2). Participants in generations 2–10 observed the classifications of a randomly sampled member of the previous generation and could choose to draw additional samples as many times as they wished.

At the beginning of the experiment, the true classifications for each task were chosen uniformly at random. To reduce variance between networks, the lab test results for a given classification were the same for all participants in a single generation (see Fig. 2). The probability of positive evidence in a lab test result was 0.6 for true classifications and 0.3 for false classifications (participants were informed of both probabilities).

### 3.2 Results and discussion

We limited our analysis to participants in generations 2–10 and preregistered our statistical analyses and sample size before the experiment.^3^ These analyses, however, were adjusted and expanded over the course of revisions. This included adding model comparisons, and using all the test data in the regression described below (rather than only one decision in generations 6–10) for consistency with our analysis for Experiment 2. Limiting our regression to the preregistered subset does not change our findings.

**Fig. 3:**
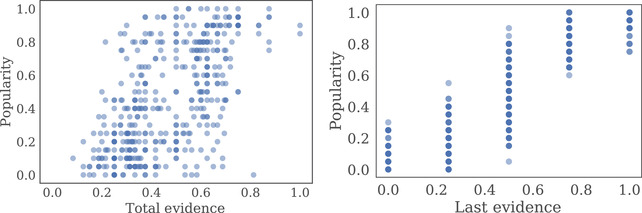
Experiment 1 results. The graph on the left shows the relationship between the total evidence available for each gem classification and the proportion of participants who selected that classification (i.e., the popularity of the classification).The graph on the right plots the relationship between the most recently observed evidence and the popularity. Each point represents a gem classification for a single shift, trial, and replication.

As predicted by our social sampling model, we observed a strong correlation between the popularity of each gem classification and both the total evidence (r(430)=0.6,p<.0001) and the most recent evidence (r(430)=0.88,p<.0001; see Fig. 3). Furthermore, total evidence was significantly predictive of popularity in a regression that included the total evidence, most recent evidence, and fixed effects for each network (t(424)=4.54,p<.0001; see Table 1 for full regression results). The correlation between popularity and total evidence shows that the proportion of social sampling was high enough to facilitate significant information aggregation over time.

**Table 1:** Regression results for Experiments 1 and 2

	Regression Model	
Variable	Experiment 1	Experiment 2
Intercept	−0.10**	0.29***
	(−3.15)	(12.69)
Total evidence	0.24***	0.05*
	(4.54)	(2.44)
Last evidence	0.91***	0.25***
	(29.26)	(18.74)
Dependent variable	Popularity	Popularity
Degrees of freedom	424	2270
Observations	432	2304

*t* statistics are in parentheses.

*p<.05, **p<.01, ***p<.001.

Note that fixed effects for each network (Experiments 1 and 2) and each condition (Experiment 2) were included in the regressions but are not shown.

We then compared the performance of our Bayesian social sampling model to three asocial models and two social models—a probability matching social sampling model and naive copying model (models are described in detail in the SEM). In the probability matching social sampling model, individuals accept candidate hypotheses with probability equal to the fraction of most recent positive evidence and in the naive copying model, individuals simply copy others' categorizations. To account for non‐social learners, both social sampling models include a term capturing the proportion of social decision making. We estimated this value as the proportion of trials where participants chose to resample at least once and found participants did so on 19% of trials. This is a lower bound on the true proportion of social sampling, since participants may have used the initial social information without resampling. However, it is close to both a separate estimate of the asocial learning proportion based on a qualitative coding of participants' strategy descriptions, and the estimated regression coefficient on total evidence available, which should correspond to the level of social sampling (see Table 1).

**Fig. 4:**
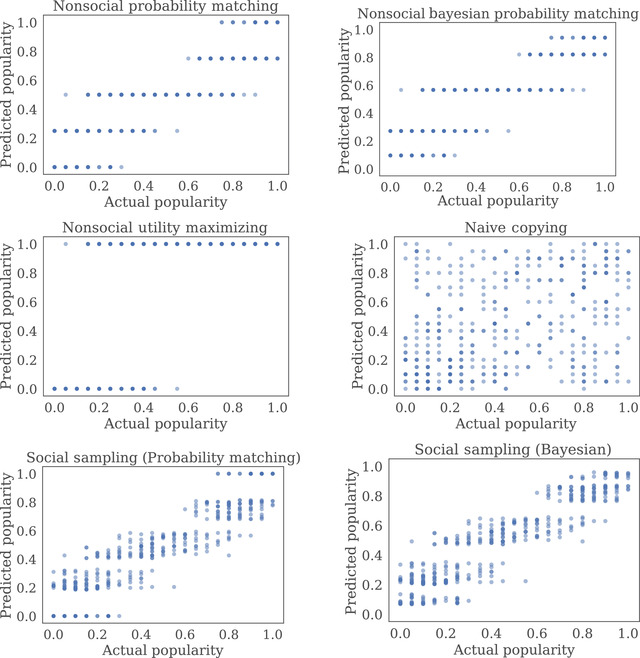
Model predictions for the three non‐social models and three social models we compared in Experiment 1. As in Fig. 3, 432 points are shown in each plot.

No free parameters are used in any of these models, and so we evaluate each model using their performance on all the experimental data. We found that the probability matching social sampling model achieved the lowest mean squared error (MSE) in predicting the popularity of each gem classification (MSE:0.0.0181), followed closely by the Bayesian social sampling model (MSE:0.0.0182; see Fig. 4 and Table 2 for results). Because both social sampling models predict accumulation, these results support our other analyses from Experiment 1, suggesting that networks of participants accumulated information about each categorization across time.

**Table 2:** Mean squared error of predicted and observed popularity for each model in Experiment 1

Model	Mean Squared Error
Non‐social problem matching	0.0213
Non‐social Bayesian problem matching	0.0220
Non‐social utility maximizing	0.1220
Naive copying	0.1260
Social sampling (problem matching)	**0.0181**
Social sampling (Bayesian)	0.0182

The best performing model is shown in bold.

## 4 Experiment 2: Spaceship construction

We constructed Experiment 1 to have a high correspondence with the assumptions of our social sampling model. Experiment 2 was designed to test our model in a less idealized context. Most notably, in Experiment 2l all information was social—participants received feedback on the *previous generation's* decisions, rather than their own. Furthermore, we did not allow participants to resample from the previous generation, and feedback was censored based on the observed participant's choices. That is, participants did not always observe evidence on every possible categorization.

**Fig. 5:**
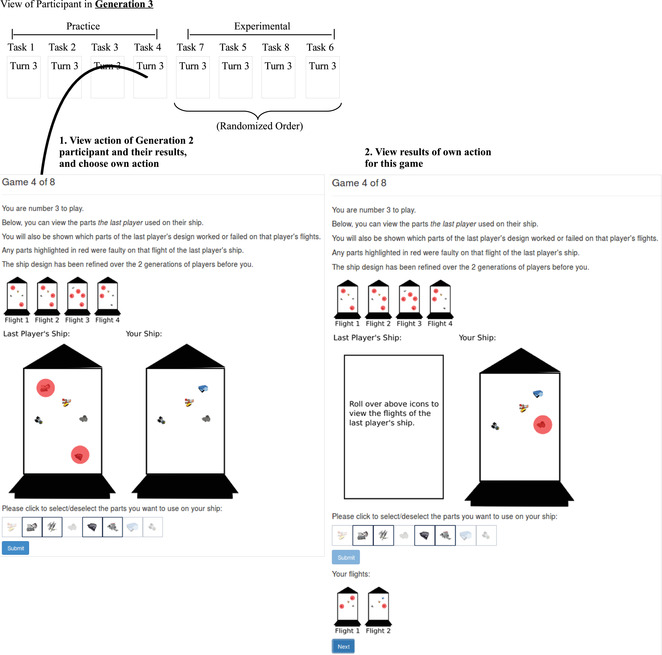
Example of the participant interface for the Experiment 2 spaceship design task. Each trial was associated with a different set of spaceship parts (using different visual icons). Participants were organized into discrete generations, or “turns.” In each generation after the first, a participant started by viewing an example spaceship design from a participant of the last generation. The participant then chose their own parts for that task and observed the part failures on each flight of that design.

In Experiment 2, participants designed spaceships by choosing which components to include from an inventory of alternatives (see Fig. 5).^4^ Each component was either a good part that rarely failed or a bad part that failed often. On each trial, participants observed spaceships designed in the previous generation, along with the success or failure of each of the included components on one or more flights. We organized participants into five conditions, varying the number of participants in each generation (large vs. small), the amount of evidence presented to each participant (high vs. low), and the strength of that evidence (high vs. low) (see Table 3).

**Table 3:** The parameter settings associated with each condition of Experiment 2

Experiment	Reps	N	J	θ
Condition 1	2	20	4	0.6
Condition 2	1	20	1	0.6
Condition 3	1	20	4	0.8
Condition 4	2	5	4	0.6
Condition 5	2	5	1	0.6

*Note*. The repetitions (reps) is the number of repetitions of the parameter settings we ran. N is the number of participants per generation. J is the number of flights, that is the amount of evidence, shown per spaceship design trial. θ gives the probability of success of good parts, and the probability of failure of bad parts—that is, the strength of evidence.

### 4.1 Methods

#### 4.1.1 Participants

We recruited 1000 participants from Amazon Mechanical Turk. Recruitment was restricted to participants living in the United States. The task typically took participants around 5–10 min to complete, and participants earned $1.80 as compensation.

#### 4.1.2 Stimuli

Experiment 2 consisted of a spaceship design categorization task inspired by prior experiments on social learning (Brand, Brown, & Cross, 2018). Example stimuli from this categorization task are shown in Fig. 5. In this task, participants designed spaceships by selecting which parts to include from a set of available options. A different set of eight parts was available on each trial. Each of the eight items in a trial's set of spaceship parts could be included in that trial's spaceship design. Participants could select or remove a part in a trial's design by clicking on an icon of the part.

Participants were organized into 10 discrete generations, with each generation composed of a different set of participants. In the first generation, each participant had no information about part failures and had to simply guess which parts might be good or bad. After selecting which parts to include, a participant was given feedback from a number of “flights” of their constructed spaceship. On each flight, spaceships parts could either succeed or fail. Each flight was shown in sequence, and we did not allow participants to change the spaceship design between flights.

In all generations except the first, participants viewed the spaceship design and flight outcomes from a spaceship selected uniformly at random from the designs made in the previous generation. Participants viewing another participant's design could select which of the prior flights to examine, as different parts may have failed on different flights. A prior design was displayed in the same way that a participant's own spaceship design was displayed, except that parts could not be modified on a previous design and part failures were shown at the start of the trial. Unlike Experiment 1, participants could not resample a choice from the previous generation.

#### 4.1.3 Procedure

Participants completed eight categorization tasks. The first four trials participants completed were practice trials, and the last four were test trials. Practice trials were displayed in the same order for all participants, and test trials were ordered using simple randomization. We did not inform participants of this practice‐test distinction, and we limited our analysis to data from the four test trials.

**Fig. 6:**
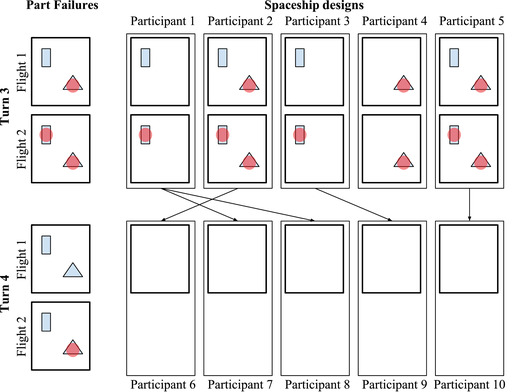
Illustration of the evidence constraints in Experiment 2. As in Experiment 1, evidence was correlated across participants: If a part failed on one flight, it failed for all participants in that generation on that flight (part failures are displayed in the figure as red circles). In contrast to Experiment 1, participants could not change their spaceship designs in between flights, and evidence about a particular spaceship part was only shown if the part was chosen in the sample design shown to the participant.

In order to mirror the structure of the social sampling model, we recruited participants in discrete generations. Generation t was recruited after all participants in generation t−1 completed the experiment. In the first generation, good and bad parts were chosen at random with probability .5. As in Experiment 1, spaceship failures were perfectly correlated across designs for a single flight in a given generation to reduce variance between networks. That is, if two different participants both used the same part in a certain generation, then the part would either succeed or fail for both participants on a given flight (see Fig. 6).

#### 4.1.4 Results and discussion

As in Experiment 1, all statistical tests were two‐tailed with an alpha level of 0.05 and we excluded the initial generation of participants from our analysis. We ran experiment conditions separately, and so no between‐subject randomization into conditions was used. Instead, participants could only participate in the experiment once and thus could not complete multiple experimental conditions.

**Fig. 7:**
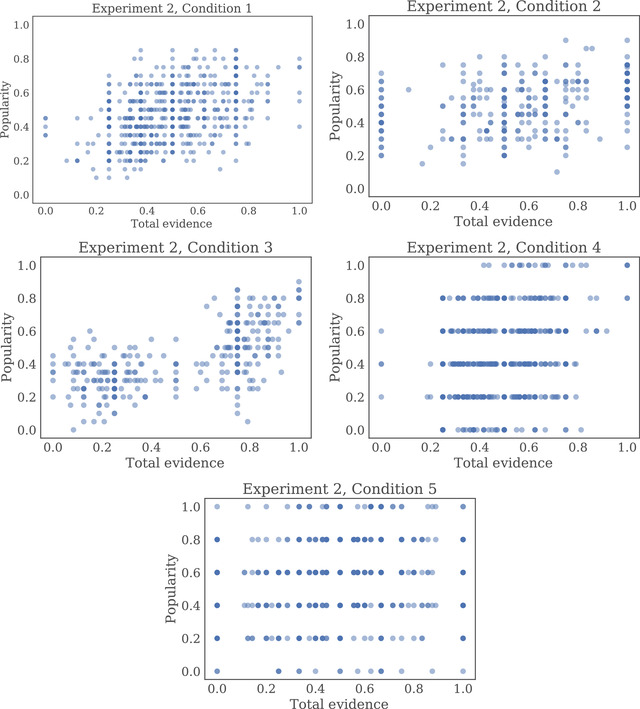
Plots showing the relationship between the total evidence available for each particular spaceship part and the proportion of participants who selected that part in Experiment 2. Each point represents one part (of eight) in one social generation (of nine) on one trial (of four) in a single repetition.

Replicating our findings from Experiment 1, we found that total evidence was significantly predictive of popularity, controlling for the most recent evidence observed in a regression that included the total evidence, most recent evidence, and fixed effects for each network and condition (t(2270)=2.44,p=.015; full results in Table 1). While we found significant positive correlations between popularity and total evidence in each of our conditions, the strength of this correlation varied between 0.16 in Condition 5 and 0.65 in Condition 3 (see Table 4 and Fig. 7). Indeed, popularity tended to be less correlated with total evidence in networks with small generation sizes, low evidence strength, and low evidence amount.

**Table 4:** Overview of the five conditions of Experiment 2 and the correlation between popularity and total evidence within each condition

Condition	Reps	Population Size	Evidence Amount	Evidence Strength	Correlation Between Popularity and Total Evidence
Condition 1	2	Large	High	Low	.43***
Condition 2	1	Large	Low	Low	.39***
Condition 3	1	Large	High	High	.65***
Condition 4	2	Small	High	Low	.29***
Condition 5	2	Small	Low	Low	.16***

*p<.05, **p<.01, ***p<.001.

All decisions in Experiment 2 were social, and so we limited model comparison to the three social models used in Experiment 1 and set the proportion of asocial learners to zero. As in Experiment 1, each model was assessed by comparing the popularity predicted by the model at each generation with the observed popularity. We found that in four of the five experimental conditions, the Bayesian social sampling model achieved the lowest MSE in predicting observed popularity. However, in the remaining condition, where the evidence strength was high, the naive copying model outperformed both the Bayesian and probability matching social sampling models (Bayesian social sampling MSE: 0.1188; probability matching social sampling MSE: 0.0825; naive copying MSE: 0.0371; see Table 5).

**Table 5:** Mean squared error of predicted and observed popularity for the three social models we compared for each of the conditions (C1–C5) in Experiment 2

	Mean Squared Error				
Model	C1	C2	C3	C4	C5
Naive copying	0.0433	0.0424	**0.0371**	0.1078	0.1030
Social sampling (matching)	0.0542	0.1703	0.0825	0.1069	0.2196
Social sampling (Bayesian)	**0.0366**	**0.0333**	0.1188	**0.0969**	**0.0960**

The best performing model in each column is shown in bold.

## 5 General discussion

Understanding how people make intelligent decisions under limited time and cognitive resources is a central focus of research in cognitive science, psychology, and economics. Social interactions offer a way for people to overcome these limitations by distributing computation across a group. By observing and learning from others, individuals do not need to perform complex computations or to condition on large amounts of data to make rational inferences. Instead, they can use simple heuristics that leverage accumulated social information.

To show how distributed inference can emerge in populations, we derived a social sampling model of individual decision making in multigenerational networks. We then showed that social sampling at the individual level can lead to distributed Bayesian inference at the population level. We tested the predictions of our social sampling model in two highly controlled behavioral experiments where participants made simple categorization decisions. While the transmission structure we used in both experiments was idealized and highly simplified compared to real‐world social networks, this allowed us to directly quantify the degree to which information accumulated across time relative to the Bayesian ideal.

Although we observe information accumulation in both experiments, the performance of the Bayesian social sampling model varied in different conditions. Most notably, in Experiment 1 the probability social sampling model achieved a slightly lower MSE than the Bayesian social sampling model (see Table 2). Furthermore, in Experiment 2 the naive copying model had a lower MSE than both social sampling models in large networks with high levels of strong evidence (see Tables 4 and 5). These results suggest that individuals may adapt their social learning strategies to different domains and may be more likely to use social sampling in noisy, low‐information environments (Toyokawa, Whalen, & Laland, 2018).

It is important to emphasize that for information aggregation to occur, the precise details of how social sampling takes place are less important than that individuals' decisions incorporate social information and new evidence using a probabilistic rule. Indeed, our social sampling models in Experiment 1 included a mixture of social and non‐social decision makers, replicating related findings on underexploitation of social information in behavioral experiments (Mercier & Morin, 2019). Our analyses are thus not intended to show that social sampling is a definitive description of human behavior, but that a social decision‐making model derived from a normative Bayesian standard can help make sense of the extent of information aggregation we observe in specific experimental contexts.

While we find that information aggregation can be robust to individual differences, accumulation can fail if people's decision‐making differs systematically from a probabilistic belief adoption–rejection strategy. Indeed, related research on the wisdom of crowds has shown that in certain contexts social interactions and observations can actually decrease group performance by reducing the diversity and independence of people's beliefs (Jenness, 1932; Lorenz, Rauhut, Schweitzer, & Helbing, 2011; Myers & Bishop, 1971). These dynamics—that is, whether social interactions improve or worsen group outcomes—appear to vary depending on people's social learning strategies (Toyokawa et al., 2018). For example, accumulation can be disrupted if people are utility‐maximizing rather than probabilistic in their responses to evidence (Anderson & Holt, 1997; Bikhchandani, Hirshleifer, & Welch, 1992). While probability matching in individual decision‐making is observed in certain domains (Shanks, Tunney, & McCarthy, 2002; Vul et al., 2014; Vulkan, 2000), our experiments show that this strategy can be extended to social settings: when a substantial proportion of individuals incorporate social information into their probability matching behavior, information accumulates across people and time. However, people may be less likely to follow a social sampling strategy in domains with low levels of probability matching, such as tasks with large financial incentives or consistent feedback (Shanks et al., 2002).

Our findings complement other approaches to understanding rationality in limited individuals. For example, previous work on adaptive heuristics (Gigerenzer & Goldstein, 1996) has shown that people use simple decision‐making rules to exploit the structure of the environment and make decisions on complex tasks. Our work suggests that this framework can be extended to social decision making. Furthermore, by providing a rational motivation for people's social heuristics, our work offers a way to connect adaptive heuristics with resource‐rational analysis (Griffiths, Lieder, & Goodman, 2015) in simple multigenerational populations. Integrating our model and findings with other frameworks for studying bounded rationality, such as models that utilize quantum probability theory (Pothos et al., 2021), should be addressed in future work.

We tested our model on a simple binary‐choice decision‐making task where the transmission structure was explicitly designed to match our model assumptions. These simplifications limit our ability to draw general conclusions about information accumulation in natural populations. For example, while enforcing uniform sampling of the previous generation gave us tight control over the transmission dynamics, people's sampling, and exploration strategies may depend on the task (Oaksford & Chater, 1994) and are likely considerably more complex in naturalistic domains and networks (Latora, Nicosia, & Russo, 2017). Similarly, people may not always transmit their true beliefs—instead, they may give noisy, incomplete, or misleading accounts of their opinions and decisions to others (Xu & Griffiths, 2010).

Despite these limitations, the general framework we have outlined and the principle of identifying the computational structure of population dynamics could therefore be extended to more complex and naturalistic domains in future work. For example, individuals could be given greater control over which (if any) beliefs to transmit or sample from others. Models of learning in structured representational domains, such as language of thought models (Goodman, Tenenbaum, & Gerstenberg, 2015) or posterior sampling in general Markov decision processes (Agrawal & Jia, 2017; Osband, Van Roy, & Russo, 2013), could also be extended to the population setting in a way that is analogous to our extension of simple categorization decisions. Testing our model and quantifying knowledge accumulation in more naturalistic domains is an exciting challenge for future work.

## Supporting information

Supporting Information

Supporting Information

## Footnotes

1 This assumption may seem implausible in large populations, but it is satisfied if each person chooses from a small number of others and those others are an unbiased sample from the full population.

2 See https://github.com/pkrafft/Overcoming‐individual‐limitations‐through‐distributed‐computation for experiment code and data.

3 Preregistration available at https://osf.io/7qx8e/?view_only=53781c6aa4c742e2a9843fcef533dfd7.

4 See https://github.com/pkrafft/Overcoming‐individual‐limitations‐through‐distributed‐computation for experiment code and data.
