# Supplementary material for: Overcoming Individual Limitations Through Distributed Computation: Rational Information Accumulation in Multigenerational Populations
Source: Top Cogn Sci. 2022 Jan 15;14(3):550–73. doi: 10.1111/tops.12596 (PMC9542743; doi:10.1111/tops.12596)
Supplement: Supplementary file 1 — Supporting Information [file TOPS-14-550-s002.pdf]

Click here to access/download;Supplementary Material;info_accumulation_SEM.pdf

Supplementary Electronic Materials for:

Overcoming individual limitations through distributed computation

Model

Here, we describe our Bayesian social sampling in detail and prove Equation 2.

Environment Model

We assume that the state of the environment which is to be inferred by the population of learners can be represented as a binary feature vector, $\boldsymbol{x}$. Features are related to observed evidence via simple logical relations. Observed pieces of evidence are represented in $\boldsymbol{Y}$. Evidence about the feature vector arrives over time, so $\boldsymbol{Y} = (\boldsymbol{y}_1, \ldots, \boldsymbol{y}_T)$, where T is the time horizon. We assume that at each time t, J new pieces of evidence about feature i are available, $y_{ijt} \sim P_i$, where P_i is a distribution specific to feature i. We assume that y_{ijt} are independent, so P_i is simply a component of a conditional probability table, $P_i = P(y_{ijt} = 1 \,|\, x_i = 0) = \theta_{i0}$ if $x_i = 0$ and $P_i = P(y_{ijt} = 1 \,|\, x_i = 1) = \theta_{i1}$ if $x_i = 1$. Under these assumptions, evidence is either perceived as positive or negative, and pieces of evidence are uncorrelated across time.

Social Sampling

To develop a normative model of behavior in this environment, we specify an individual-level, sample-based social heuristic that leads to population-rational, distributed Bayesian inference in aggregate. Crucially, group computation can emerge even when the social learning heuristic is simple and easy to compute—population rationality does not require unbounded, perfectly rational individuals. Indeed, individuals rely on social information *because of* the limited time and cognitive resources they have to make their decisions.

Our model is specified at the level of the individual in that we seek an explanation in terms of the behavioral mechanisms of individual people, and is sample-based as it relies on the assumption that people do not fully represent uncertainty in their mental representations. Instead, we assume that people often think of the world as simply being one way or the other at a particular point in time. Formally, we let N be the number of agents, d_{ait} be a discrete decision of agent a about the value of feature i at

time t, and $p_{it} = \frac{1}{N}\sum_a \mathbb{1}(d_{a,i,t-1} = 1)$ be the proportion of agents who believe $x_i = 1$ at time $t-1$. θ_{i1}, θ_{i0}, and y_{ijt} are defined as in the environment model above. For notational convenience, we also let $S_{it} = \sum_{j=1}^{J} \mathbb{1}(y_{ijt} = 1)$.

In our social sampling model, at each time step t each agent a uses a heuristic decision-making rule: The agent starts out undecided, and continues to sample possible decisions until decided. To sample a decision, agent a samples an agent a' who was decided on the last time step uniformly at random, and considers the choice of agent a', $d_{a',i,t-1}$. Agent a then decides $d_{a',i,t-1}$ with probability proportional to $(\theta_{i,d_{a',i,t-1}})^{S_{it}}(1-\theta_{i,d_{a',i,t-1}})^{J-S_{it}}$, which is the likelihood of the evidence that agent a has at time t under the state of the world indicated by agent a'. The number of samples agent a takes is a geometric random variable with parameter $p_{it} \cdot (\theta_{i1})^{nS_{it}}(1-\theta_{i1})^{J-S_{it}} + (1-p_{it}) \cdot (\theta_{i0})^{S_{it}}(1-\theta_{i0})^{J-S_{it}}$, and is finite with probability one.

Evidence Accumulation

Because each iteration of the decision loop is independent, the probability of a deciding $d_{ait} = 1$ is

$$\frac{(\theta_{i1})^{S_{it}}(1-\theta_{i1})^{J-S_{it}} \cdot p_{it}}{(\theta_{i1})^{S_{it}}(1-\theta_{i1})^{J-S_{it}} \cdot p_{it} + (\theta_{i0})^{S_{it}}(1-\theta_{i0})^{J-S_{it}} \cdot (1-p_{it})}. \tag{4}$$

In an infinite population of agents, for a particular feature i, we then have (by induction)

$$\begin{aligned}
p_{i,t+1} &= \frac{(\theta_{i1})^{S_{it}}(1-\theta_{i1})^{J-S_{it}} \cdot p_{it}}{(\theta_{i1})^{S_{it}}(1-\theta_{i1})^{J-S_{it}} \cdot p_{it} + (\theta_{i0})^{S_{it}}(1-\theta_{i0})^{J-S_{it}} \cdot (1-p_{it})} \\
&= \frac{P(\boldsymbol{y}_{i,\cdot,t} \,|\, x_i = 1) \cdot p_{it}}{P(\boldsymbol{y}_{i,\cdot,t} \,|\, x_i = 1) \cdot p_{it} + P(\boldsymbol{y}_{i,\cdot,t} \,|\, x_i = 0) \cdot (1-p_{it})} \\
&= \frac{P(\boldsymbol{y}_{i,\cdot,t} \,|\, x_i = 1) P(x_i = 1 \,|\, \boldsymbol{y}_{i,\cdot,<t})}{P(\boldsymbol{y}_{i,\cdot,t} \,|\, x_i = 1) P(x_i = 1 \,|\, \boldsymbol{y}_{i,\cdot,<t}) + P(\boldsymbol{y}_{i,\cdot,t} \,|\, x_i = 0) P(x_i = 0 \,|\, \boldsymbol{y}_{i,\cdot,<t})} \\
&= \frac{P(\boldsymbol{y}_{i,\cdot,\le t} \,|\, x_i = 1) P(x_i = 1)}{P(\boldsymbol{y}_{i,\cdot,\le t})} \\
&= P(x_i = 1 \,|\, \boldsymbol{y}_{i,\cdot,\le t})
\end{aligned}$$

being the posterior distribution on x given all preceding evidence.

Model comparison

We compared the performance of the Bayesian social sampling model with three alternative non-social models and two alternative social models. We assessed the fit of each model by comparing the aggregate popularity observed at each generation with the popularity predicted by the model.

Non-social Probability Matching Models

Our first two non-social models differentiate between pure probability matching and Bayesian probability matching.

Non-social Probability Matching. The non-social probability matching model describes decision-makers who make a feature categorization with probability equal to the evidence they have directly observed for that categorization being correct. In this case, the predicted popularity of a categorization is the fraction of positive evidence for that categorization in the current generation, $\frac{S_{it}}{J}$. If no evidence is observed, the predicted popularity of the categorization is zero.

Non-social Bayesian Probability Matching. The non-social Bayesian probability matching model describes decision-makers who make feature categorizations with probability equal to the posterior probability of the categorization being correct, given the evidence they have directly observed. The predicted popularity of a categorization is thus equal to the posterior probability for that categorization being correct given the evidence in the current generation, $P(x_{it} = 1 \mid \boldsymbol{y}_{i,\cdot,t})$.

Non-social Utility Maximizing

Our third non-social comparison model describes individuals who follow a utility maximizing strategy based on only the evidence they have directly observed. A non-social utility maximizing agent makes a feature categorization if the posterior probability for that categorization is at least 0.5. In this case

$$P(d_{ait} = 1) = \begin{cases} 1 & \text{if } P(x_{it} = 1 \,|\, \boldsymbol{y}_{i,\cdot,t}) > 0.5 \\ 0 & \text{if } P(x_{it} = 1 \,|\, \boldsymbol{y}_{i,\cdot,t}) < 0.5. \end{cases} \tag{5}$$

That is, the predicted popularity of a categorization is 1 if the posterior for that categorization is 0.5 or higher, and 0 otherwise (ties are broken randomly).

Naive Copying

Our fourth comparison model describes a purely social decision-maker who copies the categorization of a previously observed agent. In this model, the first generation makes their choices uniformly at random, and the predicted popularity of a categorization decision i at generation t is equal to p_{it}, the popularity of decision i at the previous generation.

Social Sampling Models

In addition to our hypothesized Bayesian social sampling model, we consider an alternative social sampling model that is based on pure probability matching. While both this model and the Bayesian social sampling model lead to information accumulation over time, the Bayesian social sampling model allows us to view transmission as distributed inference, offering a closer correspondence to the Bayesian approach to modeling individual cognition.

To fit both of these models, we first observed that a large proportion of participants in Experiment 1 ignored social information. To derive predictions from our social sampling models on Experiment 1, we therefore assumed that there is a mixture of non-social sampling using the corresponding probability matching model and social sampling. We let p_{ns} be the proportion of non-social sampling.

Social Sampling (Probability Matching). In the probability matching social sampling model, individuals iteratively sample decisions at random from the previous generation and accept or reject the components of those decisions based on the latest observed evidence. In this case, the acceptance probability is the same as the decision

probability in the non-social probability matching model. The predicted popularity according to this combination of components is the previous popularity of the categorization times the fraction of most recent positive evidence, normalized by the previous unpopularity of the categorization decision times the most recent negative evidence. Introducing non-social agents into this model is equivalent to setting the prior popularity to the proportion of social agents times the previous popularity of the categorization plus a uniform prior times the proportion of non-social agents. The predicted popularity at each generation is thus

$$\frac{\frac{S_{it}}{J} \cdot (p_{it} \cdot (1 - p_{ns}) + 0.5 \cdot p_{ns})}{\frac{S_{it}}{J} \cdot (p_{it} \cdot (1 - p_{ns}) + 0.5 \cdot p_{ns}) + (1 - \frac{S_{it}}{J}) \cdot ((1 - p_{it}) \cdot (1 - p_{ns}) + 0.5 \cdot p_{ns})}. \tag{6}$$

In the edge case where the popularity of a decision is equal to one but its evidence is equal to zero, the estimated popularity is set to zero.

Social Sampling (Bayesian). The Bayesian social sampling model is our primary hypothesized model that was previously described in detail. In this model, individuals iteratively sample decisions at random from the previous generation and accept or reject the components of those decisions based on the Bayesian likelihood of the categorization being correct given the latest evidence. In this model, the acceptance probability is proportional to the decision probability in the non-social Bayesian probability matching model. The predicted popularity according to this combination of components is the previous popularity of a categorization times the likelihood of the categorization being correct given the most recent evidence, normalized by the previous unpopularity of the categorization times the likelihood of the categorization being incorrect given the most recent evidence. Integrating non-social decision-makers into this model is equivalent to modeling the prior popularity as the proportion of social agents times the previous popularity of a categorization, plus the proportion of non-social agents times a uniform prior. These components yield the predicted popularity at each generation to be

$$\frac{\gamma_{it1} \cdot (p_{it} \cdot (1 - p_{ns}) + 0.5 \cdot p_{ns})}{\gamma_{it1} \cdot (p_{it} \cdot (1 - p_{ns}) + 0.5 \cdot p_{ns}) + \gamma_{it0} \cdot ((1 - p_{it}) \cdot (1 - p_{ns}) + 0.5 \cdot p_{ns})}, \tag{7}$$

where $\gamma_{itk} = \theta_{ik}^{S_{it}}(1 - \theta_{ik})^{J - S_{it}}$.

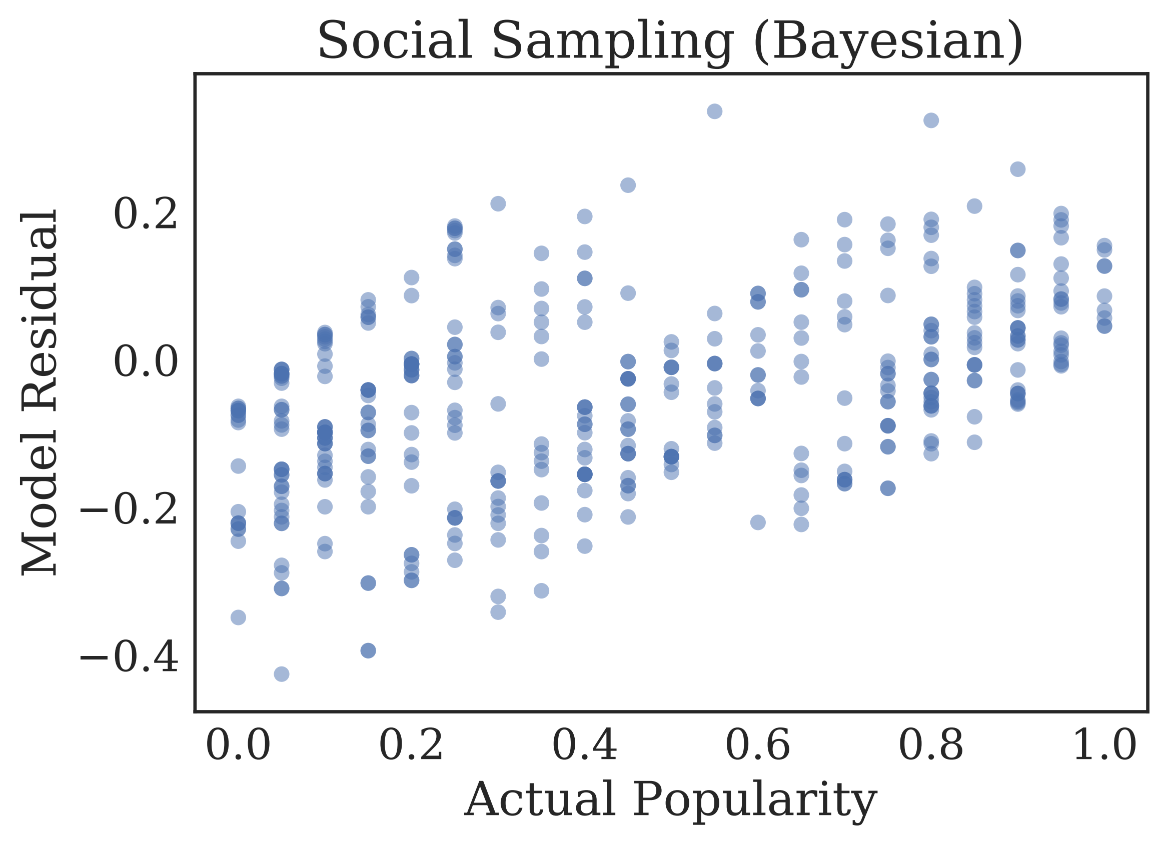
Social Sampling (Bayesian)
Model Residual
0.2
0.0
−0.2
−0.4
0.0
0.2
0.4
0.6
0.8
1.0
Actual Popularity

Click here to access/download;Supplementary Material;supplementary_material.tex

```
This is pdfTeX, Version 3.14159265-2.6-1.40.21 (TeX Live 2020/W32TeX)
(preloaded format=pdflatex 2020.5.12)  30 NOV 2021 12:32
entering extended mode
 restricted \write18 enabled.
 %&-line parsing enabled.
**supplementary_material.tex
(./supplementary_material.tex
LaTeX2e <2020-02-02> patch level 5
L3 programming layer <2020-05-05> (c:/TeXLive/2020/texmf-
dist/tex/latex/apa6/ap
a6.cls
Document Class: apa6 2020/01/13 v2.34 APA formatting (6th edition)
(c:/TeXLive/2020/texmf-dist/tex/latex/base/article.cls
Document Class: article 2019/12/20 v1.4l Standard LaTeX document class
(c:/TeXLive/2020/texmf-dist/tex/latex/base/size12.clo
File: size12.clo 2019/12/20 v1.4l Standard LaTeX file (size option)
)
\c@part=\count167
\c@section=\count168
\c@subsection=\count169
\c@subsubsection=\count170
\c@paragraph=\count171
\c@subparagraph=\count172
\c@figure=\count173
\c@table=\count174
\abovecaptionskip=\skip47
\belowcaptionskip=\skip48
\bibindent=\dimen134
) (./apacite.sty
Package: apacite 2012/02/25 v6.01 APA citation
\c@BibCnt=\count175
\bibleftmargin=\skip49
\bibindent=\skip50
\bibparsep=\skip51
\bibitemsep=\skip52
\biblabelsep=\skip53
) (c:/TeXLive/2020/texmf-dist/tex/latex/natbib/natbib.sty
Package: natbib 2010/09/13 8.31b (PWD, AO)
\bibhang=\skip54
\bibsep=\skip55
LaTeX Info: Redefining \cite on input line 694.
\c@NAT@ctr=\count176
)
LaTeX Info: Redefining \BBA on input line 213.
Class apa6 Info: The selected bibliography package, apacite and natbib,
have be
en loaded on input line 213.
(c:/TeXLive/2020/texmf-dist/tex/latex/lm/lmodern.sty
Package: lmodern 2009/10/30 v1.6 Latin Modern Fonts
LaTeX Font Info:    Overwriting symbol font `operators' in version
`normal'
(Font)                  OT1/cmr/m/n --> OT1/lmr/m/n on input line 22.
LaTeX Font Info:    Overwriting symbol font `letters' in version `normal'
(Font)                  OML/cmm/m/it --> OML/lmm/m/it on input line 23.
```

```
LaTeX Font Info:    Overwriting symbol font `symbols' in version `normal'
(Font)                  OMS/cmsy/m/n --> OMS/lmsy/m/n on input line 24.
LaTeX Font Info:    Overwriting symbol font `largesymbols' in version
`normal'
(Font)                  OMX/cmex/m/n --> OMX/lmex/m/n on input line 25.
LaTeX Font Info:    Overwriting symbol font `operators' in version `bold'
(Font)                  OT1/cmr/bx/n --> OT1/lmr/bx/n on input line 26.
LaTeX Font Info:    Overwriting symbol font `letters' in version `bold'
(Font)                  OML/cmm/b/it --> OML/lmm/b/it on input line 27.
LaTeX Font Info:    Overwriting symbol font `symbols' in version `bold'
(Font)                  OMS/cmsy/b/n --> OMS/lmsy/b/n on input line 28.
LaTeX Font Info:    Overwriting symbol font `largesymbols' in version
`bold'
(Font)                  OMX/cmex/m/n --> OMX/lmex/m/n on input line 29.
LaTeX Font Info:    Overwriting math alphabet `\mathbf' in version
`normal'
(Font)                  OT1/cmr/bx/n --> OT1/lmr/bx/n on input line 31.
LaTeX Font Info:    Overwriting math alphabet `\mathsf' in version
`normal'
(Font)                  OT1/cmss/m/n --> OT1/lmss/m/n on input line 32.
LaTeX Font Info:    Overwriting math alphabet `\mathit' in version
`normal'
(Font)                  OT1/cmr/m/it --> OT1/lmr/m/it on input line 33.
LaTeX Font Info:    Overwriting math alphabet `\mathtt' in version
`normal'
(Font)                  OT1/cmtt/m/n --> OT1/lmtt/m/n on input line 34.
LaTeX Font Info:    Overwriting math alphabet `\mathbf' in version `bold'
(Font)                  OT1/cmr/bx/n --> OT1/lmr/bx/n on input line 35.
LaTeX Font Info:    Overwriting math alphabet `\mathsf' in version `bold'
(Font)                  OT1/cmss/bx/n --> OT1/lmss/bx/n on input line 36.
LaTeX Font Info:    Overwriting math alphabet `\mathit' in version `bold'
(Font)                  OT1/cmr/bx/it --> OT1/lmr/bx/it on input line 37.
LaTeX Font Info:    Overwriting math alphabet `\mathtt' in version `bold'
(Font)                  OT1/cmtt/m/n --> OT1/lmtt/m/n on input line 38.
) (c:/TeXLive/2020/texmf-dist/tex/latex/base/fontenc.sty
Package: fontenc 2020/02/11 v2.0o Standard LaTeX package
LaTeX Font Info:    Trying to load font information for T1+lmr on input
line 11
2.
(c:/TeXLive/2020/texmf-dist/tex/latex/lm/t1lmr.fd
File: t1lmr.fd 2009/10/30 v1.6 Font defs for Latin Modern
)) (c:/TeXLive/2020/texmf-dist/tex/latex/geometry/geometry.sty
Package: geometry 2020/01/02 v5.9 Page Geometry
(c:/TeXLive/2020/texmf-dist/tex/latex/graphics/keyval.sty
Package: keyval 2014/10/28 v1.15 key=value parser (DPC)
\KV@toks@=\toks15
) (c:/TeXLive/2020/texmf-dist/tex/generic/iftex/ifvtex.sty
Package: ifvtex 2019/10/25 v1.7 ifvtex legacy package. Use iftex instead.
(c:/TeXLive/2020/texmf-dist/tex/generic/iftex/iftex.sty
Package: iftex 2020/03/06 v1.0d TeX engine tests
))
\Gm@cnth=\count177
\Gm@cntv=\count178
\c@Gm@tempcnt=\count179
```

```
\Gm@bindingoffset=\dimen135
\Gm@wd@mp=\dimen136
\Gm@odd@mp=\dimen137
\Gm@even@mp=\dimen138
\Gm@layoutwidth=\dimen139
\Gm@layoutheight=\dimen140
\Gm@layouthoffset=\dimen141
\Gm@layoutvoffset=\dimen142
\Gm@dimlist=\toks16
) (c:/TeXLive/2020/texmf-dist/tex/latex/graphics/graphicx.sty
Package: graphicx 2019/11/30 v1.2a Enhanced LaTeX Graphics (DPC,SPQR)
(c:/TeXLive/2020/texmf-dist/tex/latex/graphics/graphics.sty
Package: graphics 2019/11/30 v1.4a Standard LaTeX Graphics (DPC,SPQR)
(c:/TeXLive/2020/texmf-dist/tex/latex/graphics/trig.sty
Package: trig 2016/01/03 v1.10 sin cos tan (DPC)
) (c:/TeXLive/2020/texmf-dist/tex/latex/graphics-cfg/graphics.cfg
File: graphics.cfg 2016/06/04 v1.11 sample graphics configuration
)
Package graphics Info: Driver file: pdftex.def on input line 105.
(c:/TeXLive/2020/texmf-dist/tex/latex/graphics-def/pdftex.def
File: pdftex.def 2018/01/08 v1.0l Graphics/color driver for pdftex
))
\Gin@req@height=\dimen143
\Gin@req@width=\dimen144
) (c:/TeXLive/2020/texmf-dist/tex/latex/booktabs/booktabs.sty
Package: booktabs 2020/01/12 v1.61803398 Publication quality tables
\heavyrulewidth=\dimen145
\lightrulewidth=\dimen146
\cmidrulewidth=\dimen147
\belowrulesep=\dimen148
\belowbottomsep=\dimen149
\aboverulesep=\dimen150
\abovetopsep=\dimen151
\cmidrulesep=\dimen152
\cmidrulekern=\dimen153
\defaultaddspace=\dimen154
\@cmidla=\count180
\@cmidlb=\count181
\@aboverulesep=\dimen155
\@belowrulesep=\dimen156
\@thisruleclass=\count182
\@lastruleclass=\count183
\@thisrulewidth=\dimen157
) (c:/TeXLive/2020/texmf-dist/tex/latex/threeparttable/threeparttable.sty
Package: threeparttable 2003/06/13  v 3.0
\@tempboxb=\box45
)
\b@level@one@skip=\skip56
\e@level@one@skip=\skip57
\b@level@two@skip=\skip58
\e@level@two@skip=\skip59
\b@level@three@skip=\skip60
\e@level@three@skip=\skip61
\b@level@four@skip=\skip62
```

```
\e@level@four@skip=\skip63
\b@level@five@skip=\skip64
\e@level@five@skip=\skip65
(c:/TeXLive/2020/texmf-dist/tex/latex/caption/caption.sty
Package: caption 2020/01/03 v3.4h Customizing captions (AR)
(c:/TeXLive/2020/texmf-dist/tex/latex/caption/caption3.sty
Package: caption3 2020/01/03 v1.8h caption3 kernel (AR)
Package caption3 Info: TeX engine: e-TeX on input line 61.
\captionmargin=\dimen158
\captionmargin@=\dimen159
\captionwidth=\dimen160
\caption@tempdima=\dimen161
\caption@indent=\dimen162
\caption@parindent=\dimen163
\caption@hangindent=\dimen164
Package caption Info: Standard document class detected.
)
\c@caption@flags=\count184
\c@continuedfloat=\count185
Package caption Info: threeparttable package is loaded.
)
\c@APAenum=\count186
\@text@par@indent=\skip66
\gr@box=\box46
\gr@boxwidth=\skip67
\gr@boxheight=\skip68
(c:/TeXLive/2020/texmf-dist/tex/latex/tools/bm.sty
Package: bm 2019/07/24 v1.2d Bold Symbol Support (DPC/FMi)
\symboldoperators=\mathgroup4
\symboldletters=\mathgroup5
\symboldsymbols=\mathgroup6
LaTeX Font Info:    Redeclaring math alphabet \mathbf on input line 141.
LaTeX Info: Redefining \bm on input line 209.
)
\c@appendix=\count187
(c:/TeXLive/2020/texmf-dist/tex/latex/fancyhdr/fancyhdr.sty
Package: fancyhdr 2019/01/31 v3.10 Extensive control of page headers and
footer
s
\f@nch@headwidth=\skip69
\f@nch@O@elh=\skip70
\f@nch@O@erh=\skip71
\f@nch@O@olh=\skip72
\f@nch@O@orh=\skip73
\f@nch@O@elf=\skip74
\f@nch@O@erf=\skip75
\f@nch@O@olf=\skip76
\f@nch@O@orf=\skip77
) (c:/TeXLive/2020/texmf-dist/tex/latex/endfloat/endfloat.sty
Package: endfloat 2019/04/15 v2.7 LaTeX2e package puts figures and tables
at en
d (jdm)
\c@posttable=\count188
\efloat@postttt=\write3
```

```
\@ef@tttopen=\count189
\c@postfigure=\count190
\efloat@postfff=\write4
\@ef@fffopen=\count191
) (c:/TeXLive/2020/texmf-dist/tex/latex/float/float.sty
Package: float 2001/11/08 v1.3d Float enhancements (AL)
\c@float@type=\count192
\float@exts=\toks17
\float@box=\box47
\@float@everytoks=\toks18
\@floatcapt=\box48
)) (c:/TeXLive/2020/texmf-dist/tex/generic/babel/babel.sty
Package: babel 2020/04/28 3.43 The Babel package
(c:/TeXLive/2020/texmf-dist/tex/generic/babel/babel.def
File: babel.def 2020/04/28 3.43 Babel common definitions
\babel@savecnt=\count193
\U@D=\dimen165
(c:/TeXLive/2020/texmf-dist/tex/generic/babel/txtbabel.def)
\bbl@readstream=\read2
)
\bbl@dirlevel=\count194
(c:/TeXLive/2020/texmf-dist/tex/generic/babel-english/english.ldf
Language: english 2017/06/06 v3.3r English support from the babel system
Package babel Info: \l@canadian = using hyphenrules for english
(babel)             (\language0) on input line 102.
Package babel Info: \l@australian = using hyphenrules for ukenglish
(babel)             (\language21) on input line 105.
Package babel Info: \l@newzealand = using hyphenrules for ukenglish
(babel)             (\language21) on input line 108.
)) (c:/TeXLive/2020/texmf-dist/tex/latex/base/inputenc.sty
Package: inputenc 2018/08/11 v1.3c Input encoding file
\inpenc@prehook=\toks19
\inpenc@posthook=\toks20
(c:/TeXLive/2020/texmf-dist/tex/latex/ucs/utf8x.def
File: utf8x.def 2004/10/17 UCS: Input encoding UTF-8
)) (c:/TeXLive/2020/texmf-dist/tex/latex/ucs/ucs.sty
Package: ucs 2013/05/11 v2.2 UCS: Unicode input support
(c:/TeXLive/2020/texmf-dist/tex/latex/ucs/data/uni-global.def
File: uni-global.def 2013/05/13 UCS: Unicode global data
)
\uc@secondtry=\count195
\uc@combtoks=\toks21
\uc@combtoksb=\toks22
\uc@temptokena=\toks23
) (c:/TeXLive/2020/texmf-dist/tex/latex/parskip/parskip.sty
Package: parskip 2020-01-22 v2.0d non-zero parskip adjustments
(c:/TeXLive/2020/texmf-dist/tex/latex/kvoptions/kvoptions.sty
Package: kvoptions 2019/11/29 v3.13 Key value format for package options
(HO)
(c:/TeXLive/2020/texmf-dist/tex/generic/ltxcmds/ltxcmds.sty
Package: ltxcmds 2019/12/15 v1.24 LaTeX kernel commands for general use
(HO)
) (c:/TeXLive/2020/texmf-dist/tex/generic/kvsetkeys/kvsetkeys.sty
Package: kvsetkeys 2019/12/15 v1.18 Key value parser (HO)
```

```
)) (c:/TeXLive/2020/texmf-dist/tex/latex/etoolbox/etoolbox.sty
Package: etoolbox 2019/09/21 v2.5h e-TeX tools for LaTeX (JAW)
\etb@tempcnta=\count196
)) (c:/TeXLive/2020/texmf-dist/tex/latex/url/url.sty
\Urlmuskip=\muskip16
Package: url 2013/09/16  ver 3.4  Verb mode for urls, etc.
) (c:/TeXLive/2020/texmf-dist/tex/latex/tools/hhline.sty
Package: hhline 2020/01/04 v2.04 Table rule package (DPC)
) (c:/TeXLive/2020/texmf-dist/tex/latex/tools/array.sty
Package: array 2019/08/31 v2.4l Tabular extension package (FMi)
\col@sep=\dimen166
\ar@mcellbox=\box49
\extrarowheight=\dimen167
\NC@list=\toks24
\extratabsurround=\skip78
\backup@length=\skip79
\ar@cellbox=\box50
) (c:/TeXLive/2020/texmf-dist/tex/latex/makecell/makecell.sty
Package: makecell 2009/08/03 V0.1e Managing of Tab Column Heads and Cells
\rotheadsize=\dimen168
\c@nlinenum=\count197
\TeXr@lab=\toks25
) (c:/TeXLive/2020/texmf-dist/tex/latex/xcolor/xcolor.sty
Package: xcolor 2016/05/11 v2.12 LaTeX color extensions (UK)
(c:/TeXLive/2020/texmf-dist/tex/latex/graphics-cfg/color.cfg
File: color.cfg 2016/01/02 v1.6 sample color configuration
)
Package xcolor Info: Driver file: pdftex.def on input line 225.
Package xcolor Info: Model `cmy' substituted by `cmy0' on input line
1348.
Package xcolor Info: Model `hsb' substituted by `rgb' on input line 1352.
Package xcolor Info: Model `RGB' extended on input line 1364.
Package xcolor Info: Model `HTML' substituted by `rgb' on input line
1366.
Package xcolor Info: Model `Hsb' substituted by `hsb' on input line 1367.
Package xcolor Info: Model `tHsb' substituted by `hsb' on input line
1368.
Package xcolor Info: Model `HSB' substituted by `hsb' on input line 1369.
Package xcolor Info: Model `Gray' substituted by `gray' on input line
1370.
Package xcolor Info: Model `wave' substituted by `hsb' on input line
1371.
)
\@input{macros.aux}
\openout2 = `macros.aux'.

(./macros.tex (c:/TeXLive/2020/texmf-
dist/tex/latex/algorithmicx/algpseudocode.
sty
Package: algpseudocode
(c:/TeXLive/2020/texmf-dist/tex/latex/base/ifthen.sty
Package: ifthen 2014/09/29 v1.1c Standard LaTeX ifthen package (DPC)
) (c:/TeXLive/2020/texmf-dist/tex/latex/algorithmicx/algorithmicx.sty
Package: algorithmicx 2005/04/27 v1.2 Algorithmicx
```

```
Document Style algorithmicx 1.2 - a greatly improved `algorithmic' style
\c@ALG@line=\count198
\c@ALG@rem=\count199
\c@ALG@nested=\count266
\ALG@tlm=\skip80
\ALG@thistlm=\skip81
\c@ALG@Lnr=\count267
\c@ALG@blocknr=\count268
\c@ALG@storecount=\count269
\c@ALG@tmpcounter=\count270
\ALG@tmplength=\skip82
)
Document Style - pseudocode environments for use with the `algorithmicx'
style
) (c:/TeXLive/2020/texmf-dist/tex/latex/algorithms/algorithm.sty
Package: algorithm 2009/08/24 v0.1 Document Style `algorithm' - floating
enviro
nment
\@float@every@algorithm=\toks26
\c@algorithm=\count271
) (c:/TeXLive/2020/texmf-dist/tex/latex/mathtools/mathtools.sty
Package: mathtools 2020/03/24 v1.24 mathematical typesetting tools
(c:/TeXLive/2020/texmf-dist/tex/latex/tools/calc.sty
Package: calc 2017/05/25 v4.3 Infix arithmetic (KKT,FJ)
\calc@Acount=\count272
\calc@Bcount=\count273
\calc@Adimen=\dimen169
\calc@Bdimen=\dimen170
\calc@Askip=\skip83
\calc@Bskip=\skip84
LaTeX Info: Redefining \setlength on input line 80.
LaTeX Info: Redefining \addtolength on input line 81.
\calc@Ccount=\count274
\calc@Cskip=\skip85
) (c:/TeXLive/2020/texmf-dist/tex/latex/mathtools/mhsetup.sty
Package: mhsetup 2017/03/31 v1.3 programming setup (MH)
) (c:/TeXLive/2020/texmf-dist/tex/latex/amsmath/amsmath.sty
Package: amsmath 2020/01/20 v2.17e AMS math features
\@mathmargin=\skip86
For additional information on amsmath, use the `?' option.
(c:/TeXLive/2020/texmf-dist/tex/latex/amsmath/amstext.sty
Package: amstext 2000/06/29 v2.01 AMS text
(c:/TeXLive/2020/texmf-dist/tex/latex/amsmath/amsgen.sty
File: amsgen.sty 1999/11/30 v2.0 generic functions
\@emptytoks=\toks27
\ex@=\dimen171
)) (c:/TeXLive/2020/texmf-dist/tex/latex/amsmath/amsbsy.sty
Package: amsbsy 1999/11/29 v1.2d Bold Symbols
LaTeX Info: Redefining \boldsymbol on input line 28.
\pmbraise@=\dimen172
) (c:/TeXLive/2020/texmf-dist/tex/latex/amsmath/amsopn.sty
Package: amsopn 2016/03/08 v2.02 operator names
)
\inf@bad=\count275
```

```
LaTeX Info: Redefining \frac on input line 227.
\uproot@=\count276
\leftroot@=\count277
LaTeX Info: Redefining \overline on input line 389.
\classnum@=\count278
\DOTSCASE@=\count279
LaTeX Info: Redefining \ldots on input line 486.
LaTeX Info: Redefining \dots on input line 489.
LaTeX Info: Redefining \cdots on input line 610.
\Mathstrutbox@=\box51
\strutbox@=\box52
\big@size=\dimen173
LaTeX Font Info:    Redeclaring font encoding OML on input line 733.
LaTeX Font Info:    Redeclaring font encoding OMS on input line 734.
\macc@depth=\count280
\c@MaxMatrixCols=\count281
\dotsspace@=\muskip17
\c@parentequation=\count282
\dspbrk@lvl=\count283
\tag@help=\toks28
\row@=\count284
\column@=\count285
\maxfields@=\count286
\andhelp@=\toks29
\eqnshift@=\dimen174
\alignsep@=\dimen175
\tagshift@=\dimen176
\tagwidth@=\dimen177
\totwidth@=\dimen178
\lineht@=\dimen179
\@envbody=\toks30
\multlinegap=\skip87
\multlinetaggap=\skip88
\mathdisplay@stack=\toks31
LaTeX Info: Redefining \[ on input line 2859.
LaTeX Info: Redefining \] on input line 2860.
)
LaTeX Info: Thecontrolsequence`\('isalreadyrobust on input line 130.
LaTeX Info: Thecontrolsequence`\)'isalreadyrobust on input line 130.
LaTeX Info: Thecontrolsequence`\['isalreadyrobust on input line 130.
LaTeX Info: Thecontrolsequence`\]'isalreadyrobust on input line 130.
\g_MT_multlinerow_int=\count287
\l_MT_multwidth_dim=\dimen180
\origjot=\skip89
\l_MT_shortvdotswithinadjustabove_dim=\dimen181
\l_MT_shortvdotswithinadjustbelow_dim=\dimen182
\l_MT_above_intertext_sep=\dimen183
\l_MT_below_intertext_sep=\dimen184
\l_MT_above_shortintertext_sep=\dimen185
\l_MT_below_shortintertext_sep=\dimen186
\xmathstrut@box=\box53
\xmathstrut@dim=\dimen187
) (c:/TeXLive/2020/texmf-dist/tex/latex/bbm-macros/bbm.sty
Package: bbm 1999/03/15 V 1.2 provides fonts for set symbols - TH
```

```
LaTeX Font Info:    Overwriting math alphabet `\mathbbm' in version
`bold'
(Font)                  U/bbm/m/n --> U/bbm/bx/n on input line 33.
LaTeX Font Info:    Overwriting math alphabet `\mathbbmss' in version
`bold'
(Font)                  U/bbmss/m/n --> U/bbmss/bx/n on input line 35.
)

! LaTeX Error: Command \bm already defined.
               Or name \end... illegal, see p.192 of the manual.

See the LaTeX manual or LaTeX Companion for explanation.
Type  H <return>  for immediate help.
 ...

l.27 \newcommand{\bm}{\boldsymbol{m}}

Your command was ignored.
Type  I <command> <return>  to replace it with another command,
or  <return>  to continue without it.


! LaTeX Error: Option clash for package inputenc.

See the LaTeX manual or LaTeX Companion for explanation.
Type  H <return>  for immediate help.
 ...

l.95 \usepackage
                [english]{babel}
The package inputenc has already been loaded with options:
  [utf8x]
There has now been an attempt to load it with options
  [utf8]
Adding the global options:
  utf8x,utf8
to your \documentclass declaration may fix this.
Try typing  <return>  to proceed.

(c:/TeXLive/2020/texmf-dist/tex/latex/amscls/amsthm.sty
Package: amsthm 2017/10/31 v2.20.4
\thm@style=\toks32
\thm@bodyfont=\toks33
\thm@headfont=\toks34
\thm@notefont=\toks35
\thm@headpunct=\toks36
\thm@preskip=\skip90
\thm@postskip=\skip91
\thm@headsep=\skip92
\dth@everypar=\toks37
)
\c@definition=\count288
\c@theorem=\count289
\c@lemma=\count290
```

```
\c@proposition=\count291
\c@corollary=\count292
) (c:/TeXLive/2020/texmf-dist/tex/latex/letltxmacro/letltxmacro.sty
Package: letltxmacro 2019/12/03 v1.6 Let assignment for LaTeX macros (HO)
) (c:/TeXLive/2020/texmf-dist/tex/latex/xcite/xcite.sty
Package: xcite 2020/01/21 v16383.99998 eXternal Citations (EG)
(c:/TeXLive/2020/texmf-dist/tex/latex/tools/xr.sty
Package: xr 2019/07/22 v5.05 eXternal References (DPC)
))
Package xr Info: IMPORTING LABELS FROM main.aux on input line 59.
(main.tex)
(main.aux)
(c:/TeXLive/2020/texmf-dist/tex/latex/l3backend/l3backend-pdfmode.def
File: l3backend-pdfmode.def 2020-05-05 L3 backend support: PDF mode
\l__kernel_color_stack_int=\count293
\l__pdf_internal_box=\box54
) (./supplementary_material.aux)
\openout1 = `supplementary_material.aux'.

LaTeX Font Info:    Checking defaults for OML/cmm/m/it on input line 61.
LaTeX Font Info:    ... okay on input line 61.
LaTeX Font Info:    Checking defaults for OMS/cmsy/m/n on input line 61.
LaTeX Font Info:    ... okay on input line 61.
LaTeX Font Info:    Checking defaults for OT1/cmr/m/n on input line 61.
LaTeX Font Info:    ... okay on input line 61.
LaTeX Font Info:    Checking defaults for T1/cmr/m/n on input line 61.
LaTeX Font Info:    ... okay on input line 61.
LaTeX Font Info:    Checking defaults for TS1/cmr/m/n on input line 61.
LaTeX Font Info:    ... okay on input line 61.
LaTeX Font Info:    Checking defaults for OMX/cmex/m/n on input line 61.
LaTeX Font Info:    ... okay on input line 61.
LaTeX Font Info:    Checking defaults for U/cmr/m/n on input line 61.
LaTeX Font Info:    ... okay on input line 61.
\c@maskedRefs=\count294
(c:/TeXLive/2020/texmf-dist/tex/latex/apacite/english.apc
File: english.apc 2013/07/21 v6.03 apacite language file
LaTeX Info: Redefining \BPBI on input line 129.
LaTeX Info: Redefining \BHBI on input line 130.
)
*geometry* driver: auto-detecting
*geometry* detected driver: pdftex
*geometry* verbose mode - [ preamble ] result:
* driver: pdftex
* paper: a4paper
* layout: <same size as paper>
* layoutoffset:(h,v)=(0.0pt,0.0pt)
* modes: twoside
* h-part:(L,W,R)=(72.26999pt, 452.9679pt, 72.26999pt)
* v-part:(T,H,B)=(72.26999pt, 700.50687pt, 72.26999pt)
* \paperwidth=597.50787pt
* \paperheight=845.04684pt
* \textwidth=452.9679pt
* \textheight=700.50687pt
* \oddsidemargin=0.0pt
```

```
* \evensidemargin=0.0pt
* \topmargin=-37.0pt
* \headheight=15.2pt
* \headsep=25.0pt
* \topskip=12.0pt
* \footskip=30.0pt
* \marginparwidth=95.0pt
* \marginparsep=10.0pt
* \columnsep=10.0pt
* \skip\footins=10.8pt plus 4.0pt minus 2.0pt
* \hoffset=0.0pt
* \voffset=0.0pt
* \mag=1000
* \@twocolumnfalse
* \@twosidetrue
* \@mparswitchtrue
* \@reversemarginfalse
* (1in=72.27pt=25.4mm, 1cm=28.453pt)

(c:/TeXLive/2020/texmf-dist/tex/context/base/mkii/supp-pdf.mkii
[Loading MPS to PDF converter (version 2006.09.02).]
\scratchcounter=\count295
\scratchdimen=\dimen188
\scratchbox=\box55
\nofMPsegments=\count296
\nofMParguments=\count297
\everyMPshowfont=\toks38
\MPscratchCnt=\count298
\MPscratchDim=\dimen189
\MPnumerator=\count299
\makeMPintoPDFobject=\count300
\everyMPtoPDFconversion=\toks39
) (c:/TeXLive/2020/texmf-dist/tex/latex/epstopdf-pkg/epstopdf-base.sty
Package: epstopdf-base 2020-01-24 v2.11 Base part for package epstopdf
Package epstopdf-base Info: Redefining graphics rule for `.eps' on input
line 4
85.
(c:/TeXLive/2020/texmf-dist/tex/latex/latexconfig/epstopdf-sys.cfg
File: epstopdf-sys.cfg 2010/07/13 v1.3 Configuration of (r)epstopdf for
TeX Liv
e
)) (c:/TeXLive/2020/texmf-dist/tex/latex/apa6/config/APAamerican.txt
File: APAamerican.txt 2012/02/23 v1.25 apa6 configuration for American
English
)
Package caption Info: Begin \AtBeginDocument code.
Package caption Info: float package is loaded.
Package caption Info: End \AtBeginDocument code.
(c:/TeXLive/2020/texmf-dist/tex/latex/ucs/ucsencs.def
File: ucsencs.def 2011/01/21 Fixes to fontencodings LGR, T3
) [1
```

```
{c:/TeXLive/2020/texmf-var/fonts/map/pdftex/updmap/pdftex.map}]
LaTeX Font Info:    Trying to load font information for OT1+lmr on input
line 7
9.
(c:/TeXLive/2020/texmf-dist/tex/latex/lm/ot1lmr.fd
File: ot1lmr.fd 2009/10/30 v1.6 Font defs for Latin Modern
)
LaTeX Font Info:    Trying to load font information for OML+lmm on input
line 7
9.
(c:/TeXLive/2020/texmf-dist/tex/latex/lm/omllmm.fd
File: omllmm.fd 2009/10/30 v1.6 Font defs for Latin Modern
)
LaTeX Font Info:    Trying to load font information for OMS+lmsy on input
line
79.
(c:/TeXLive/2020/texmf-dist/tex/latex/lm/omslmsy.fd
File: omslmsy.fd 2009/10/30 v1.6 Font defs for Latin Modern
)
LaTeX Font Info:    Trying to load font information for OMX+lmex on input
line
79.
(c:/TeXLive/2020/texmf-dist/tex/latex/lm/omxlmex.fd
File: omxlmex.fd 2009/10/30 v1.6 Font defs for Latin Modern
)
LaTeX Font Info:    External font `lmex10' loaded for size
(Font)              <12> on input line 79.
LaTeX Font Info:    External font `lmex10' loaded for size
(Font)              <8> on input line 79.
LaTeX Font Info:    External font `lmex10' loaded for size
(Font)              <6> on input line 79.
LaTeX Font Info:    Trying to load font information for U+bbm on input
line 117
.
(c:/TeXLive/2020/texmf-dist/tex/latex/bbm-macros/ubbm.fd
File: ubbm.fd 1999/03/15 V 1.2 Font definition for bbm font - TH
)
! Undefined control sequence.
<argument> \@shorttitle

l.121

The control sequence at the end of the top line
of your error message was never \def'ed. If you have
misspelled it (e.g., `\hobx'), type `I' and the correct
spelling (e.g., `I\hbox'). Otherwise just continue,
and I'll forget about whatever was undefined.

[2]
! Undefined control sequence.
<argument> \@shorttitle

l.170
```

```
The control sequence at the end of the top line
of your error message was never \def'ed. If you have
misspelled it (e.g., `\hobx'), type `I' and the correct
spelling (e.g., `I\hbox'). Otherwise just continue,
and I'll forget about whatever was undefined.

[3]
Underfull \vbox (badness 2495) has occurred while \output is active []

! Undefined control sequence.
<argument> \@shorttitle

l.193 \end{equation}

The control sequence at the end of the top line
of your error message was never \def'ed. If you have
misspelled it (e.g., `\hobx'), type `I' and the correct
spelling (e.g., `I\hbox'). Otherwise just continue,
and I'll forget about whatever was undefined.

[4]
! Undefined control sequence.
<argument> \@shorttitle

l.210 \begin{equation}

The control sequence at the end of the top line
of your error message was never \def'ed. If you have
misspelled it (e.g., `\hobx'), type `I' and the correct
spelling (e.g., `I\hbox'). Otherwise just continue,
and I'll forget about whatever was undefined.

[5] AED endfloat: Processing end Figures and Tables
! Undefined control sequence.
<argument> \@shorttitle

l.231 \end{document}

The control sequence at the end of the top line
of your error message was never \def'ed. If you have
misspelled it (e.g., `\hobx'), type `I' and the correct
spelling (e.g., `I\hbox'). Otherwise just continue,
and I'll forget about whatever was undefined.

[6] (./supplementary_material.aux) )
Here is how much of TeX's memory you used:
 9059 strings out of 480681
 129461 string characters out of 5908536
 420043 words of memory out of 5000000
 24686 multiletter control sequences out of 15000+600000
 571097 words of font info for 52 fonts, out of 8000000 for 9000
 1141 hyphenation exceptions out of 8191
 49i,13n,51p,1153b,256s stack positions out of
5000i,500n,10000p,200000b,80000s
```

```
{c:/TeXLive/2020/texmf-dist/fonts/enc/dvips
/lm/lm-rm.enc}{c:/TeXLive/2020/texmf-dist/fonts/enc/dvips/lm/lm-
mathit.enc}{c:/
TeXLive/2020/texmf-dist/fonts/enc/dvips/lm/lm-mathsy.enc}
<c:/Users/asynch/.tex
live2020/texmf-
var/fonts/pk/ljfour/public/bbm/bbm12.600pk>{c:/TeXLive/2020/texm
f-dist/fonts/enc/dvips/lm/lm-mathex.enc}{c:/TeXLive/2020/texmf-
dist/fonts/enc/d
vips/lm/lm-ec.enc}<c:/TeXLive/2020/texmf-
dist/fonts/type1/public/lm/lmbx12.pfb>
<c:/TeXLive/2020/texmf-
dist/fonts/type1/public/lm/lmex10.pfb><c:/TeXLive/2020/t
exmf-dist/fonts/type1/public/lm/lmmi12.pfb><c:/TeXLive/2020/texmf-
dist/fonts/ty
pe1/public/lm/lmmi6.pfb><c:/TeXLive/2020/texmf-
dist/fonts/type1/public/lm/lmmi8
.pfb><c:/TeXLive/2020/texmf-
dist/fonts/type1/public/lm/lmmib10.pfb><c:/TeXLive/
2020/texmf-dist/fonts/type1/public/lm/lmr12.pfb><c:/TeXLive/2020/texmf-
dist/fon
ts/type1/public/lm/lmr6.pfb><c:/TeXLive/2020/texmf-
dist/fonts/type1/public/lm/l
mr8.pfb><c:/TeXLive/2020/texmf-
dist/fonts/type1/public/lm/lmri12.pfb><c:/TeXLiv
e/2020/texmf-
dist/fonts/type1/public/lm/lmsy10.pfb><c:/TeXLive/2020/texmf-dist/
fonts/type1/public/lm/lmsy6.pfb><c:/TeXLive/2020/texmf-
dist/fonts/type1/public/
lm/lmsy8.pfb>
Output written on supplementary_material.pdf (6 pages, 142061 bytes).
PDF statistics:
 87 PDF objects out of 1000 (max. 8388607)
 64 compressed objects within 1 object stream
 0 named destinations out of 1000 (max. 500000)
 1 words of extra memory for PDF output out of 10000 (max. 10000000)
```

topiCS box

Click here to access/download;Supplementary Material;Note to Production topiCS Box.docx

This article is part of the topic “Extending Rationality,” Emmanuel M. Pothos and Timothy J. Pleskac (Topic Editors).

Blurb for TOC only

Click here to access/download;Supplementary Material;Griffiths Blurb.docx

Many of the problems people face are difficult to solve under the limited time and resources available to them. We show that individuals can overcome these limitations by following a simple social learning heuristic that yields distributed Bayesian inference at the population level. We test our model in two large behavioral experiments, comparing observed knowledge accumulation with the Bayesian ideal in multi-generational micro-societies.
